# Screening 89 Pesticides in Fishery Drugs by Ultrahigh Performance Liquid Chromatography Tandem Quadrupole-Orbitrap Mass Spectrometer

**DOI:** 10.3390/molecules24183375

**Published:** 2019-09-17

**Authors:** Shou-Ying Wang, Cong Kong, Qing-Ping Chen, Hui-Juan Yu

**Affiliations:** 1 Laboratory of Quality & Safety Risk Assessment for Aquatic products (Shanghai), Ministry of Agriculture and Rural Affairs, East China Sea Fisheries Research Institute, Shanghai 200090, China; 2 College of Food Science & Technology, Shanghai Ocean University, Shanghai 201306, China; 3 Key Laboratory of East China Sea Fishery Resources Exploitation, Ministry of Agriculture and Rural Affairs, East China Sea Fisheries Research Institute, Chinese Academy of Fishery Sciences, Shanghai 200090, China

**Keywords:** fishery drugs, high-resolution orbitrap mass spectrometry, pesticide, screening

## Abstract

Multiclass screening of drugs with high resolution mass spectrometry is of great interest due to its high time-efficiency and excellent accuracy. A high-scale, fast screening method for pesticides in fishery drugs was established based on ultrahigh performance liquid chromatography tandem quadrupole-Orbitrap high-resolution mass spectrometer. The target compounds - were diluted in methanol and extracted by ultrasonic treatment, and the extracts were diluted with MeOH-water (1:1, *v*/*v*) and centrifuged to remove impurities. The chromatographic separation was performed on an Accucore aQ-MS column (100 mm × 2.1 mm, 2.6 μm) with gradient elution using 0.1% formic acid in water (containing 5 mmol/L ammonium formate) and 0.1% formic acid in methanol (containing 5 mmol/L ammonium formate) in Full Scan/dd-MS2 (TopN) scan mode. A screening database, including mass spectrometric and chromatographic information, was established for identification of compounds. The screening detection limits of methods ranged between 1–500 mg/kg, the recoveries of real samples spiked with the concentration of 10 mg/kg and 100 mg/kg standard mixture ranged from 70% to 110% for more than sixty compounds, and the relative standard deviations (RSDs) were less than 20%. The application of this method showed that target pesticides were screened out in 10 samples out of 21 practical samples, in which the banned pesticide chlorpyrifos were detected in 3 out of the 10 samples.

## 1. Introduction

Aquaculture is estimated to provide half of aquatic products by 2030 from the farming of freshwater or marine areas [1]. There is inevitably going to be a need for intensive aquaculture developed to supply more products from this industry. According to the “Green food—fishery medicine application guideline (NY/T 755-2013)” in the Agricultural Industry Standards of the People’s Republic of China [2], fishery medicine refers to the substances that prevent or treat diseases in aquaculture animals or purposefully regulate the physiology of animals, including chemicals, antibiotics, Chinese herbal medicines and biological products. It is also known as chemical inputs or veterinary medicinal products (VMPs) applied in aquaculture in Europe and the United States [3,4]. Chemical inputs from aquaculture include antifoulants, antibiotics, parasiticides, anesthetics and disinfectants [5], while parasiticides in fishery mainly contain avermectins, pyrethroids, hydrogen peroxide, and organophosphates [5,6]. Based on the Guidelines, ten kinds of fishery drugs originated from pesticide have been banned for aquatic animals and plants. However, the illegal or excessive addition of pesticides in the fishery drug, as well as uncontrollable and uncertain administration during culture process can lead to the accumulation and residue of these pesticides in aquatic product. Illegal and unregulated use of pesticide may occur in many aquaculture areas, and further threaten the food safety for human health. To protect the quality and safety of aquatic products, as well as the sustainable ecosystem, surveillance of pesticides components in fishery drugs should be conducted.

Ultrahigh performance liquid chromatography coupled to high-resolution mass spectrometry (UHPLC-HRMS) is a promising strategy for multi-component screening of pesticides [7,8,9]. HRMS could record full scan of the precursors or fragmented ions with high-resolution, as well as the relative isotopic abundance, and is virtually able to distinguish unlimited number of compounds from one set of analyzed data [10,11]. In the past, the chromatography coupled to Time of Flight Mass Spectrometry (ToF-MS) was used in the development of multiclass components screening methods [9,12,13]. However, comparing to ToF-MS, the orbitrap mass spectrometer can fast scan and simultaneously switch between positive and negative acquisition modes if there’s no need to change mobile phase of chromatography unit [14,15]. The combination of quadrupole and Orbitrap for high-resolution mass spectrometry can acquire data with high throughput, excellent accuracy and better sensitivity, which provides an ideal platform for multiclass risk compound screening [16]. Therefore, more methods of screening detection with Orbitrap MS were developed. With this instrument, the data-dependent data acquisition mode scans the full mass distribution of all precursors and then selectively fragments them sequentially for secondary mass scanning according to their abundance. This scan mode allows the quantification of compounds with precursor ion abundancy and identification with corresponding fragment ions [17]. Moreover, due to the stable and high-resolution mass spectrum recorded at standard data provide enough dependency, the identification of targeted compound can be conducted by comparing their database rather than practically acquire data for standards every time [18,19].

In previous studies, the analysis of 139 pesticide residues in fruit and vegetable commodities was established based on the Q-Orbitrap MS, allowing the retrospective analysis of the data feature which cannot be achieved with QqQ [17]. Jia et al. have developed an untargeted screening method for 137 veterinary drugs and their metabolites (16 categories) in tilapia using UHPLC-Orbitrap MS [20]. Turnipseed established a wide-scope screening method for 70 veterinary drugs in fish, shrimp and eel using LC-Orbitrap MS [7]. Recently, a non-target data acquisition for target analysis workflow based on UHPLC/ESI Q-Orbitrap was examined for its performance in screening pesticide residues in fruit and vegetables [21]. However, there is a lack of works on the multi-component screening detection in fishery drugs, especially for pesticide component screening. A fast screening method for a wide range of pesticides detection can be preferred, as much more reagent, time, and labor can be saved to detect more harmful components for safety evaluation.

Our study aims to develop a more generic screening method for a wider scope of pesticides with a self-built database, which can keep the advantages of robustness, simplicity, and time-efficiency. In the current work, we investigated 89 possible pesticides that can be used in fishery-related industry and remained in aquatic products. The chromatographic and high-resolution mass spectra for these compounds were acquired with a UHPLC-quadrupole-Orbitrap HRMS after optimizing parameters. The useful fragment ions with high-resolution were explored and selected. Then, a database including the retention time, isotope pattern, ionization mode and adduct, characteristic fragment ions, was established. Identification rules for data comparison with real samples were also investigated. Finally, a fast pesticide screening method for fishery drug was developed in combination with a rapid pretreatment.

## 2. Results and Discussion

### 2.1. Full MS-ddMS^2^ Scan for Identification and Qualification

Full MS-ddMS^2^ detection mode was applied on UHPLC-ESI-Q-Orbitrap HRMS system, which is a different data acquisition from single (multiple) reaction monitoring on triple quadrupole mass spectrometry. The Orbitrap analyzer collected accurate mass of all precursor ions as the first identification step of compounds. The precursors of high abundance were isolated through quadrupole in the next round scanning. Each of the precursors can be fragmented sequentially in the HCD multipole, re-collected in C-trap, and analyzed through Orbitrap mass spectrometer. It should be noted that the accurate mass of precursors instead of their fragmentation ions was continuously tracked and can be integrated for peak identification. Therefore, the precursor ions can be used for quantification and their corresponding fragmented ions for each peak of precursor ion can be used for identification in combination.

Under the guideline of European SANTE/11813/2017 and Commission Decision 2002/657/EC [22], identification of the concerned analytes with high-resolution mass spectrometry can be performed. The chromatographic information, their mass information should attain given identification points (IPs) to get confirmed results. If the high-resolution mass spectrometric data were collected, 2 IPs are earned if the precursor ion match, and 2.5 IPs for each of their product ions [23,24]. For the identification of all compounds, 4.5 IPs are required. In our work, the *m*/*z* of isotope, and its relative abundance for precursors were also identified, which leads to higher IPs for structure identification. Therefore, our identification rule should be stricter and more reliable than current regulations, which can result in less false positive result according to our experiment on fortified samples.

### 2.2. Mobile Phase

**Figure 1:**
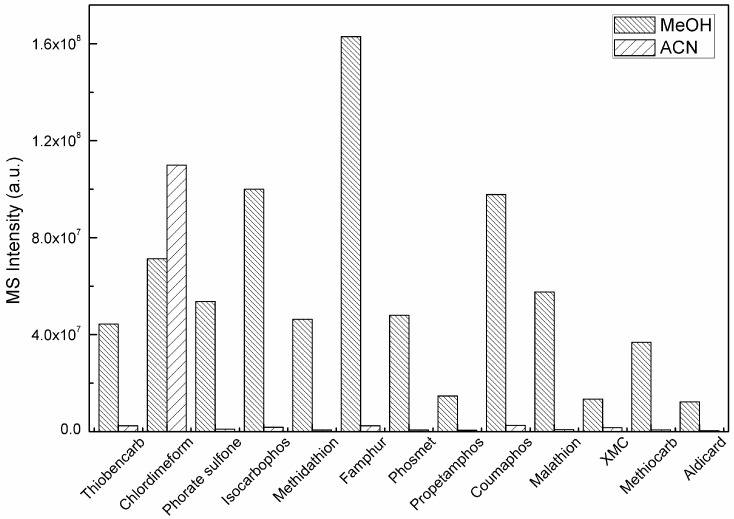
Pesticides with significant changes in sensitivity in MeOH and acetonitrile (ACN) mobile phases with 5 mM ammonium formate and 0.1% formic acid (FA) at the concentration of 50 ng/mL.

Due to the excellent performance of Accucore aQ-MS column in the analysis of multiclass compounds of different polarities, it was employed for chromatography separation of these target compounds. MeOH-water and MeCN-water binary mobile phase were investigated for the separation of the 89 compounds. In order to improve the efficiency of analyte ionization, 5 mM of ammonia formate and 0.1% formic acid (FA) were added in both phases. The result showed no triggered MS/MS spectrum for fenitrothion, chlorpyrifos, phorate, or dichlorvos since the automatic gain control AGC does not satisfy the setting value 5 × 10^5^, when MeCN was applied as mobile phase at the concentration of 50 ng/mL under the full scan/dd-MS2 acquisition, which was considered as a negative result in our experiment. Moreover, signal intensities of more than 10 compounds decreased by 1–2 orders compared with MeOH as the mobile phase. Compounds with significant difference of signal intensity are shown in Figure 1. There were unremarkable differences for the rest pesticides on either mobile phase. According to Figure 1, MeOH is a better mobile phase, as more compounds showed higher response on mass spectrometer. Therefore, MeOH-water system with buffers and formic acid was selected for eluting these compounds from the column, and which is similar to Raina’s research concerning of determination OPs in the air based on LC-MS/MS [25]. Neither MeCN nor MeOH could separate 89 pesticides completely. However, with the mass spectrometer, these compounds are not necessarily to be separated, as the different *m*/*z* can be easily acquired and extracted for different co-eluted compounds, with a pure chromatographic signal for individual compound. It should be noted that proper chromatographic elution of these compounds is still important, as it can avoid matrix effect and potentially competitive ionization between each other if high content compounds are present.

### 2.3. Buffers

**Figure 2:**
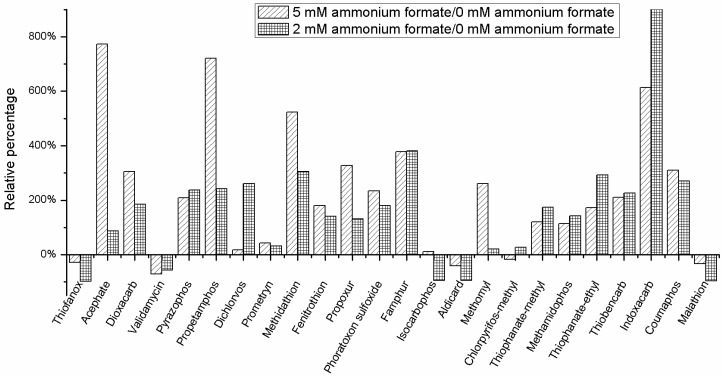
Relative signal enhancement or depression for typical compounds with different concentrations of ammonia formate or without this buffer in the mobile phase in the same gradient.

The addition of formic acid helped improve the ionization efficiency and further increased the sensitivity of analytes, which has been validated in our optimization work. In our research, different concentration of buffers (ammonium formate, 0 mM, 2 mM, and 5 mM) in mobile phase with 0.1% FA were examined for 50 ng/mL mix standards solutions in the same gradient elution. Results showed better chromatographic peaks for most of the compounds when 2 or 5 mM ammonium formate was added in the mobile phase. As it is shown in Figure 2, signals were enhanced by approximately 10 times for propetamphos, famphur, methidathion, and indoxacarb are obtained when buffers were used in the mobile phase. Furthermore, the retention time of some compounds have been delayed after addition of 5 mM ammonium formate in the mobile phase. Buffers are beneficial to the retention and separation for many compounds, especially for acephate, propetamphos, methomyl, and indoxacard, and they further increase the sensitivity, even though a soft/lower intensity on mass spectrum was shown for phorate, dichlorvos, and chlorpyrifos-methyl when 5 mM ammonium formate added. As a result, 5 mM of ammonium formate was added in both mobile phases.

### 2.4. Mass Spectrometry

In principle, the higher the resolution of mass spectrum, the identification for target compounds is more accurate. A resolution of 140,000 can be achieved with Orbitrap in our work. However, the analyze time for each scan would be extended significantly and result in a lower data sampling rate. Therefore, enough information for peak integration or critical fragments of precursors will be compromised, as there are only around 15 s of elution time for each compound in chromatography. Similar to our previous work for veterinary drug screening [26,27], full scan/dd-MS2 (TopN) was applied for mass data acquisition, in which an inclusion list of the target compounds was preset. The MS resolution for full scan and fragment acquisition are 70,000 and 17,500, respectively. It could allow the discrimination of low abundant ions undetectable under low resolution [28], and further minimize possibility of false positive [8,29]. For dd-MS2 acquisition, if the high abundant ions were preset in the inclusion list, they were fragmented and scanned sequentially once their precursors were detected. Based on the set parameters, the probability that the instrument fails to trigger MS/MS spectrum acquisition for a detected chemical is greatly reduced. No false negative results were determined for any analyte spiked above its SDL. If there were compounds showing no fragmentation acquisition at the lower concentration, which can be identified by precursor *m*/*z* abundance greater than 5 × 10^5^, isotope abundance and retention times with narrower deviation to avoid false negatives. Otherwise the compound is counted as undetected. In this work, the top 2 abundant ions were successively fragmented and transferred into the Orbitrap for data acquisition. Under the electrospray ionization, 76 of these compounds formed precursor ions as [M + H]^+^, 8 of these compounds ionized as [M + Na]^+^, and 5 pyrethroids formed additions as [M + NH_4_]^+^. PCP Na and 4 phenylpyrazoles formed negative ions as [M − H]^−^. Three different normalized collision energy (stepped NCE) allowed the high-efficiency fragmentation of different precursors at their best.

### 2.5. Sample Preparation

**Figure 3:**
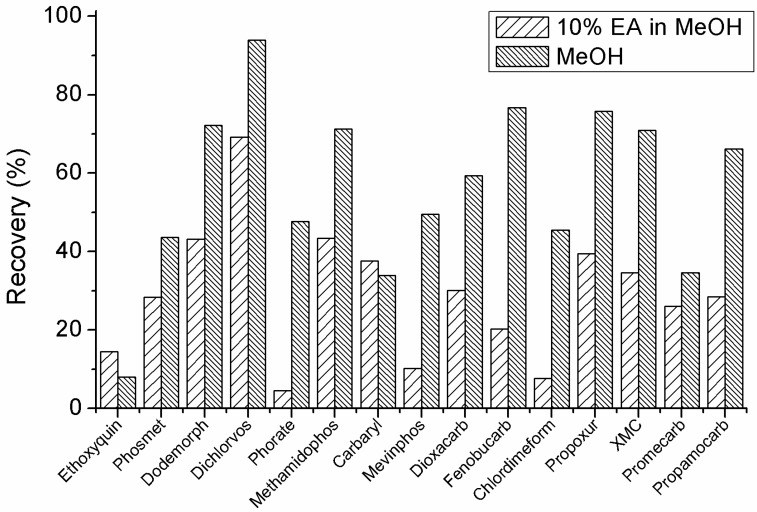
Recoveries of typical compounds with or without the use of 10% ethyl acetate in the methanol.

It is critical for high recovery determination to choose the solvent of extraction. In this research, pesticides of interest are of multiclass and of quite different chemical or physical properties. To dissolve or extract different analytes with high or low polarity, MeOH and 10% ethyl acetate in MeOH were used as extract solvents for pure Chinese herb drugs, which contains complex matrices and impurities. Results showed better extracting efficiency when MeOH was used. In terms of the recovery of these target compounds, more than half of targets showed better recovery than 10% ethyl acetate in MeOH. As it is shown in Figure 3, seven compounds including phorate, mevinphos, fenobucarb, chlordimeform, propoxur, XMC, and propamocarb showed more than 35% decrease of recovery. Therefore, MeOH was preferred as a solvent for the analysis of pesticides in these drugs.

### 2.6. Matrix Effect

Matrix effect should be considered in the detection process, which includes intrinsic organic or inorganic compounds after extraction and cleanup, and extrinsic inorganic ions, organic acids, detergent, etc. These interfere material can comprehensively enhance or suppress the response of the target compounds. In our research, the matrix effect (*ME*%) was calculated based on the following equation [30,31]:

$$ME\%=\left(\frac{A}{B}-1\right)\times 100\%$$

where *A* is the integration area in matrix-matched standard solution and *B* is the integration area in a standard solution with identical concentration for each compound. In general, the matrix effect within ±20% can be regarded as acceptable and the calibration can be performed without considering matrix effect. Otherwise, it should be considered during quantification [16,32].

The fishery drugs were dissolved and diluted up to 1000 times, which would significantly decrease the matrix effect. Standards diluted with more than 90% blank matrix solution were used to test for the matrix effect. The result showed less than 20% matrix effect for all the compounds of interest at the concentrations of 100 and 500 ng/mL for compounds with SDL above 100 ng/mL. Because of the acceptable matrix effect, it is feasible to use a methanol–water (1:1, *v*/*v*) solution to dilute a series of standard solutions, for quantification of positive compounds.

### 2.7. Method Validation

#### 2.7.1. Screening Detection Limit

According to SANTE/11813/2017 [33], the screening detection limit (SDL) was examined with similar process, but less replicates, which has been applied in many reported works [34,35,36]. Fishery drug of Pure Chinese herb was fortified with mixed standard solutions at different concentrations in six duplicates together with their non-spiked counterparts, which were used for the examination of the screening detection limit, and all compounds satisfied 100% detection criterion at their SDL. Simultaneously, an additional criterion, identity confirmation through the 13C/12C-ratio, was satisfied for each target compound at the corresponding theoretical SDL [34,35,36]. In our experiment, 1 mg/kg, 10 mg/kg, 50 mg/kg, 100 mg/kg, and 500 mg/kg of these mix target samples were prepared respectively. All these fortified samples were pretreated following the aforementioned method (2.4). Results showed that 54, 80, 85, 86, and 89 compounds were screened positive at 1 mg/kg, 10 mg/kg, 50 mg/kg, 100 mg/kg, and 500 mg/kg, respectively.

#### 2.7.2. Accuracy and Repeatability

The accuracy and repeatability of the screening method were investigated under the fortified concentrations of 10 mg/kg and 100 mg/kg in fishery drug of pure Chinese herb. For compounds at the detection limit of 500 ng/mL on the mass spectrometer, fortified samples of 500 mg/kg were prepared independently. Under the fortified concentration of 10 mg/kg and 100 mg/kg, compounds with the instrument detection limit of 10 ng/mL and below can be readily detected. Over sixty compounds showed the recovery of 70%–110% at spiked 10 and 100mg/kg; fifteen compounds with 110%–120% at 10 mg/kg; twelve compounds with 110%–120% at 100 mg/kg; and three compounds including chlorpyrifos, phosmet, and tributylphos-phorotrithioate had recoveries of over 125% at both spiked levels. Over 95% of compounds identified at both fortified levels had RSD of less than 15%. Compounds were not identified at the lower fortified level but detected at 100 mg/kg including amitraz, phorate, fenitrothion, validamycin, and prothiofos, with the recovery of 59.3%–125% and RSD of 6.17%–14.7%. Compounds only detected at the spiked level of 500 mg/kg are bromophos ethyl, cyfluthrin, parathion, with recovery of 85.3%–105% and RSD of less than 20%. All the quantification results were obtained with less than 20% RSD. Because of the soft matrix enhancement, there were some compounds with high recoveries at both fortified levels for quantification with the standard matched solvent, especially for chlorpyrifos, phosmet, and tributylphos-phorotrithioate. The details of recovery and RSD are presented in Table 1. It is noticed that some compounds did not meet the recovery criteria at one or both of the fortified levels, which could be attributed to high volatility and easy converting properties.

#### 2.7.3. Calibration and linearity

**Figure 4:**
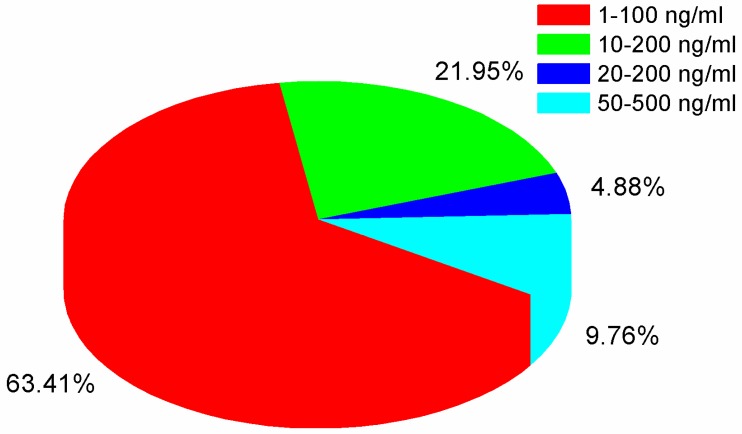
The percentages of different linear range of 82 targeted pesticides.

As the matrix effect on the response of the fishery drug sample is quite low, and the recovery results satisfied the semiquantification analysis for most of the compounds in positive samples, the standard solution without matrix matched, and internal standards can be amenable for calibration of positive samples from the perspective of economic costs. In our research, different concentrations of mixed pesticide standards were prepared directly with MeOH–water (1:1, *v*/*v*). Results on mass spectrometer demonstrated that the R-squared of 81 pesticides were no less than 0.990, and 5 other pesticides, including chlorpyrifos, flumethrin, flucythrinate, tau-fluvalinate, and deltamethrin showed R-square between 0.982 and 0.990. The detailed linear profile for 82 compounds is listed in the electronic Supplementary Material (Table S1). The distribution pie chart of the linear range of these compounds is presented in Figure 4.

### 2.8. Practical Screening

**Figure 5:**
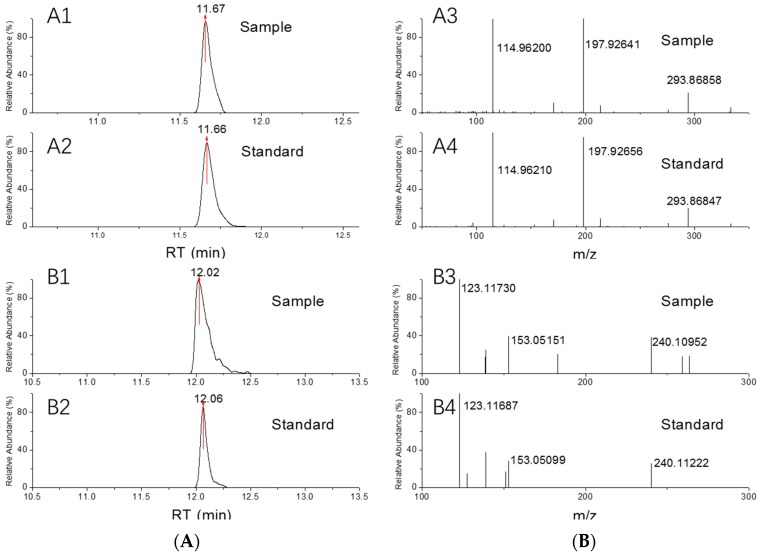
Comparison of the spectra of chlorpyrifos (**A**) and ivermectin B1a (**B**) detected in the positive samples. A1, B1 and A2, B2 are the chromatogram of real samples and standards, respectively. A3, B3 and A4, B4 is the MS/MS spectrum of the positive sample and the standard respectively.

The method was further applied in screening of 21 fishery drug samples (pesticides, water-clean agents and antibacterial agents). Samples were prepared according to sample preparation (4.4) prior to analysis. For the compounds with concentration out of the linear range, samples were re-diluted with the dilution factor of samples adjusted to ensure the concentration to be quantitatively evaluated based on our linear range. The screening was carried out following the home-built database and preset rules. Quantification was conducted through the peak areas of precursor ions in positive samples and was externally calibrated. Based on the database and preset identification rules, 10 out of the 21 fishery drug samples were screened positive with pesticides. 10 samples were detected with unspecified components. As is shown in Table 2, the identified pesticides were chlorpyrifos, ivermectin B1a, phoxim, avermectin B1a, and carbendazim. Three samples contained forbidden drug chlorpyrifos (Figure 5A), and 5 samples contain avermectin and ivermectin (Figure 5B) of more than 3 g/L. Their chromatographic and fragment information was highly identical to the standards, as are shown in Figure 5. Detailed information of the screened positive samples was presented in Table 2.

## 3. Materials and Methods

### 3.1. Instruments and Reagents

The ultrahigh-performance liquid chromatography (UHPLC) system (Dionex UltiMate 3000, Thermo Fisher Scientific, San-Francisco, USA) coupled to quadrupole Orbitrap mass spectrometer with electrospray ionization (Q-Exactive, Thermo Fisher Scientific) was used for data acquisition.

Eight-nine Pesticides were selected for target screening as listed in Table 3. Carbofuran and dichlorvos were obtained from MANHAGE Biotech. Inc. (Beijing, China), thiofanox-sulfone, thiometon, aldicarb-sulfone, phoratoxon sulfoxide, PCP Na were purchased from Accustandard Inc. (New Haven, CT, USA) The other 85 pesticides standards were supplied by Dr. Ehrenstorfer GmbH (Augsburg, Germany). Acetonitrile (MeCN) and Methanol (MeOH) of HPLC grade were obtained from J.T. Baker (Phillipsburg, NJ, USA). Formic acid (FA, 98%, LC-MS grade, Fisher Scientific, Spain, or HPLC grade) was obtained from FLUKA. All the other relevant reagents were purchased from common domestic suppliers. Pure water was obtained through Water Milli Q ELEMENT purification unit (Millipore, Bedford, USA).

### 3.2. Preparation of Standards

Standards stock solution: c.a. 5 mg solid standards was dissolved with MeOH in 10 mL beaker, and then transferred to a 50 mL flask and diluted with MeOH. For compounds will less solubility in MeOH, 0.1 mL formic acid (98%, HPLC grade) was firstly added and the mixture was sonicated until the solids were completely dissolved. Five microliters of liquid standards were pipetted into a 10 mL-beaker and weighed to get accurate mass. After that, they were dissolved in methanol following similarly procedure as the solid standards. All these single standards stock solutions were c.a. 100 μg/mL. The purchased standards solutions were not diluted until further preparation of mixed standards solution. Mixed standards solutions were prepared by mixing standards of the same category, which were finally diluted to 5 μg/mL. The standards were categorized into organophosphorus, carbamate, organochlorine, imidazole, pyrethroid, triazole, phenylpyrazole, avermectin, and miscellaneous. All the standards solutions were stored in refrigerator at −42 °C.

Matrix-matched standards were used to evaluate the matrix effect, where the standards were dissolved into a matrix of Chinese herbal fishery drug, which was negative for pesticides before spiking.

### 3.3. Methods

#### 3.3.1. Elution Conditions

Accucore aQ-MS column (2.1 × 100 mm, 2.6 μm, Thermo Fisher Scientific, USA), was employed to perform sample separation with a thermostat at 30 °C. The binary mobile phases (MP) were 0.1% FA in water (containing 5 mM Ammonium Formate, A) and 0.1% FA in MeOH (containing 5 mM Ammonium Formate, B). Their gradient elution was started with 2% B, linearly increasing to 20% in 4 min and continuously ramped to 40% within 1.5 min. Subsequentially, B was increased to 98% in the subsequent 5 min, and kept for 2.4 min. Then, B was restored to the initial conditions 2%B in the following 2.1 min, and kept for 5 min to re-equilibrate for the next injection. The whole elution process for one injection analysis took 20 min. The flow rate was kept at 0.3 mL·min^−1^. The injection volume for analysis was 10 μL for each sample.

#### 3.3.2. Mass Spectrometer Condition

Parameters for electrospray were as following: spray voltage, 3200 V (positive mode), 2800 V (negative mode), sheath gas flow rate at 40 L·min^−1^, auxiliary gas flow rate at 10 L·min^−1^, sweep gas flow rate at 1.0 L·min^−1^, auxiliary gas temperature at 350 °C, capillary temperature at 325 °C and S-lens RF level at 60 V. The scan mode for high-resolution mass spectrometry acquisition was Full MS/dd-MS2 (with inclusion list) mode. Recorded mass range for full mass record was between *m*/*z* 100–1000 (positive mode) and 150–1000 (negative mode), at resolution of 70,000. The Full MS/dd-MS2 (with inclusion list) mode can simultaneously record the precursor mass and the MS/MS (fragmentation) spectra for selected precursors. The MS/MS acquisition for fragment scanning of the selected ions was carried out at the isolation window of 2.0 *m*/*z* and the resolution of 15,000. For each round of fragmentation acquisition, the top 2 (TopN, 2, loop count 1) abundant precursors above the threshold 5 × 10^5^ were sequentially transferred into the C-Trap (AGC, 5 × 10^5^, Max IT, 100 ms) for collision at normalized energies (NCE, 20, 50, 80) in HCD multipole and pumped to Orbitrap for MS/MS acquisition.

All the units of UHPLC-ESI-Q-Orbitrap HRMS system were controlled through the Tracefinder software.

### 3.4. Sample Preparation

A 20 mL centrifuge tube was filled with 100 mg samples and added with 20 mL MeOH. One hundred microliters of liquid sample was pipetted directly into another centrifuge tube. The tube was vortexed for 1 min, and ultrasonicated for 15 min. Then, the solution was vortexed again and silenced for 2 min. Then, MeOH-water (1:1, *v*/*v*) was used to dilute 0.5 mL of supernatant by 5 fold. After vortex and silence, 1 mL of the solution was transferred to an Eppendorf tube (1.5 mL) and centrifuged at 10,000 rpm/min for 15 min to remove the precipitate. The upper supernatant was transferred into a vial for analysis.

Chinese herbal drugs were used for method validation in the research, which was representative of a complex matrix of fishery products. Fishery drugs of pure Chinese herb products composed of granular herbal extract were purchased from a local fishery store.

### 3.5. Database for Screening, Qualitative and Quantitative Rules

The names, categories, CAS numbers, formulas, expected mass of the suspected compounds were searched and collected to establish a basic database. Then, the standard solution of each compound (100 ng·mL^−1^) were analyzed through the UHPLC-ESI-Q-Orbitrap HRMS system using the aforementioned parameters. Therefore, *m*/*z* of precursor ion, retention time (RT) and fragment ions (FI) were acquired by experiments. In parallel, the isotope pattern for each precursor was automatically calculated by Tracefinder software. All information was organized and built in Tracefinder. It was used to perform screening according to the database with the following screening rules: *m*/*z* deviation of precursor ion was 3 × 10^−6^, allowed RT deviation was ±15 s, at least one fragment ion match with allowed *m*/*z* deviation at 2 × 10^−5^, and the fit threshold for precursor isotope pattern was more than 75% with allowed mass deviation within 10 ppm, and allowed isotope intensity deviation of less than 25%. If the screening rules passed for a compound, it was qualitatively identified as positive. Furthermore, a series of mixed standards solution of 1–500 ng/mL were prepared for quantification of the positive compounds. The integrated peak area of precursor ions for positive compounds was used for external calibration and quantification. The instrument detection limit (LOD), the minimum concentration that the compound could be identified under the qualitative rules, was tested at the optimized parameters. The detailed information for these compounds of interest in the database is shown in Table 3.

## 4. Conclusions

In summary, a database of 89 pesticides was built, including both chromatographic and HRMS information. The data was acquired after parameter optimization on ultrahigh performance liquid chromatography interfaced quadrupole-Orbitrap mass spectrometer. Based on the database, the screening rule for these compounds was further established by comparing their precursors, fragments, retention time and isotopes. The fast, high-throughput identification and rough quantification of these compounds was achieved. The method was successfully applied for the pesticides risk assessment of fishery drugs. However, as the detection mode established on potential and known pesticides, where their chromatographic and mass spectrometric information were examined and collected, the unknown, non-target risk compounds were ignored. Further work will be focused on non-target screening based on characteristic fragments for recognizing and monitoring risk factors. Overall, our current method can be used as a fast, reliable, efficient and practical tools for the fishery drug risk assessment, which saves more time, and expenses.

## Figures and Tables

**Table 1:** Recovery and relative standard deviation (RSD) of each drug at different spike levels in feedstuff matrices.

Compound	10 mg·kg^−1^		100 mg·kg^−1^		Compound	10 mg·kg^−1^		100 mg·kg^−1^	
	Recovery (%)	RSD (%, *n* = 3)	Recovery (%)	RSD (%, *n* = 3)		Recovery (%)	RSD (%, *n* = 3)	Recovery (%)	RSD (%, *n* = 3)
Aminocarb	104	5.99	109	4.35	2,3,5-Trimethacarb	80	1.78	103	0.71
Carbaryl	81.8	3.91	99.9	8.49	3,4,5-Trimethylphenol	86.4	4.62	101	2.91
Carbendazim	120	3.77	85.6	4.04	Acephate	102	4.75	116	4.83
Carbofuran	92.1	6.42	94	3.82	Aldicarb sulfone	94.7	10.2	103	4.07
Chlordimeform	81.7	4.96	85	3.17	Aldicarb sulfoxide	86.6	6.19	103	5.97
Coumaphos	105	7.77	109	8.11	Aldicard	89.9	18.2	105	2.97
Dimethoate	110	11.1	107	10.7	Avermectin B1a	91.4	7.87	84.5	4.24
Dodemorph	95.2	7.08	82.1	2.96	Bendiocarb	80.7	3.5	88.9	7.96
Famphur	113	7.1	111	5.44	Bifenthrin	86.5	21	86.7	5.1
Fenobucarb	82.3	3.33	105	2.89	Chlorpyrifos-methyl	94.5	23	124	10.1
Fuberidazole	111	5.44	85	4.01	Deltamethrin	77.2	9.61	79.2	4.54
Imazalil	92.6	5.67	82.2	4.31	Dichlorvos	113	7.61	117	7.23
Indoxacarb	84.2	7.71	72.9	4.11	Dioxacarb	96	5.6	104	5.34
Isocarbophos	96.9	12.3	104	8.42	Doramectin	75.1	8.76	86.6	4.26
Malathion	88	6.52	113	6.66	Ethoxyquin	94.4	5.48	30.2	16.5
Methiocarb	86.1	8.01	104	5.37	Fenvalerate	90.5	21.6	78.8	1.83
Mevinphos	84.8	10.2	115	1.98	Fipronil	80.8	9.54	89.3	2.1
Monocrotophos	113	5.67	121	4.98	Fipronil-desulfinyl	88	8.67	80.8	6.54
Omethoate	123	12.7	117	12.7	Fipronil-sulfide	83.1	8.68	86.6	3.43
PCP Na	83.1	7.62	88	0.989	Fipronil-sulfone	82.9	6.88	80.8	4.43
Phoratoxon sulfoxide	92.2	14	110	4.59	Flucythrinate	97.9	7.69	80	2.09
Phosalone	105	7.34	103	4.11	Flumethrin	65.3	5.66	77.9	0.732
Phoxim	111	10	102	7.12	Isoprocarb	80	1.78	103	0.71
Pirimicarb	85.5	7.96	99.7	2.84	Ivermectin B1a	59.7	4.64	86.3	1.29
Pirimiphos-methyl	113	8.83	119	3.26	Methamidophos	108	6.42	124	6.08
Promecarb	89.1	5.83	102	2.98	Methidathion	98	4.15	113	4.73
Prometryn	88.9	7.63	78.8	0.618	Methomyl	94	6.7	111	4.89
Propamocarb	96.3	8.1	109	5.37	Phorate sulfone	97.3	5.51	107	1.58
Propazine	93.1	8.22	72.5	2.52	Propetamphos	113	5.06	123	4.27
Propiconazole	115	6.92	77.3	0.198	Robenidine	90	12.2	64.5	1.73
Propoxur	85.2	4.67	91.2	6.94	Tau-fluvalinate	65.5	13.6	83.6	4.04
Pyrazophos	119	12.1	122	6.95	Thiofanox	80.6	2.06	95.4	3.46
Quinalphos	113	8.76	112	5.45	Thiofanox sulphone	87.7	9.49	92.4	0.0845
Simazine	86.7	3.21	65.7	1.7	Thiofanox -sulphoxide	82.9	3.47	101	2.42
Simetryne	98.2	4.56	76	3.28	XMC	75.6	9.88	85.8	6.46
Thiabendazole	119	6.16	80.9	3.43	Xylazine	90.5	8.82	76.8	1.8
Thiobencarb	117	6.87	76.8	1.54	Amitraz	-—	-—	61.9	14.2
Triazophos	112	5.76	116	3.33	Fenitrothion	-—	-—	124	13.8
trichlorfon	109	4.85	109	6.27	Phorate	—	—	125	12.1
Chlorpyrifos	138	7.17	154	7.63	Prothiofos	-—	-—	100	14.7
Phorate sulfoxide	109	9.55	126	3.67	Validamycin	—	—	59.3	6.17
Phosmet	131	10.5	177	17	Bromophos ethyl *	—	—	85.3	19.4
Thiophanate-ethyl	128	5.49	76.9	1.36	Cyfluthrin *	—	—	91.5	1.13
Thiophanate-methyl	128	4.96	77.9	1.86	Parathion *	-—	-—	105	3.53
Tributylphos-phorotrithioate	150	7.98	147	4.56					

* fortified at 500 mg/kg.

**Table 2:** Screening results of practical fishery drugs.

Code	Trade Name	Dosage Form	Listed	Detected Compounds	Contents (mg/kg or mg/L)
4	Insecticide for fish	Aqueous solution	NA	Chlorpyrifos	2.66
5	Insecticide for fish and shrimp	Soluble concentrate	Avermectin	Ivermectin B1a	347
				Chlorpyrifos	1.33
				Avermectin B1a	7479
6	Pesticide for water	Soluble concentrate	Bioactive ingredient	Ivermectin B1a	207
				Avermectin B1a	3482
14	Insecticide for fish	Soluble concentrate	NA	Phoxim	2.20
15	Avermectin solution	Soluble concentrate	Avermectin	Avermectin B1a	5937
16	Benzalkonium Bromide Solution	Aqueous solution	NA	Avermectin B1a	55,587
17	Beta-Cypermethrin Solution	Aqueous solution	Cypermethrin	Chlorpyrifos	9.11
20	Insecticide for water	Soluble concentrate	Bioactive ingredient	Ivermectin B1a	8214
21	Pesticide for water	Gel solution	Avermectin	Ivermectin B1a	121
				Avermectin B1a	3736
22	Insecticide for water	Soluble concentrate	Avermectin	Phoxim	19.7
				Avermectin B1a	1931

NA: not available. Listed: active compounds were listed in the label of fishery drugs.

**Table 3:** Chromatographic, mass spectrometric information and limit of detection of 89 targeted pesticides.

Compounds	RT (min)	Extracted Mass (*m*/*z*)	Fragment Ions (*m*/*z*)	LOD ng/mL	Compounds	RT (min)	Extracted Mass (*m*/*z*)	Fragment Ions (*m*/*z*)	LOD ng/mL
Bendiocarb	8.95	224.09173	167.07027/109.02841	1	Aldicarb sulfone	5.15	223.0747	86.06004/76.03930	1
Chlorpyrifos	11.75	349.93356	114.9615/197.92730	1	Aminocarb	4.39	209.12845	152.10699/137.08352	1
Coumaphos	10.96	363.02174	226.99263/306.95913	1	Carbaryl	9.27	202.08626	132.04439/124.08827	1
Dimethoate	7.44	230.0069	142.99623/170.96978	1	Carbendazim	6.3	192.07675	160.05054/132.05562	1
Dodemorph	9.78	282.27914	116.10699/98.09643	1	Carbofuran	8.94	222.11247	123.04406/165.09101	1
Famphur	9.55	326.02803	93.00999/142.99263	1	Chlordimeform	6.63	197.084	117.05730/152.02615	1
Fuberidazole	7.4	185.07094	157.07602/156.06820	1	Dioxacarb	7.46	224.09173	123.04406/167.07027	1
Isocarbophos	9.68	312.04299	269.99844/236.00554	1	Ethoxyquin	9.7	218.15394	148.07569/190.12264	1
Malathion	10.26	353.02529	195.06031/227.03238	1	Fenobucarb	10.02	208.13321	208.13321/95.04914	1
Mevinphos	8.12	225.05225	127.01547/67.01784	1	Fipronil	10.62	434.93143	329.95845/183.01646	1
Monocrotophos	6.65	224.06824	127.01547/58.02874	1	Fipronil-desulfinyl	10.54	386.96444	350.98667/281.99146	1
Omethoate	4.17	214.02974	214.02974/182.98754	1	Fipronil-sulfide	10.72	418.93651	57.9746/170.00863	1
Phorate sulfoxide	9.47	277.01502	114.96133/142.93848	1	Fipronil-sulfone	10.85	450.92634	414.94857/243.98839	1
Phoratoxon sulfoxide	7.61	261.03786	114.96133/128.97698	1	Imazalil	9.49	297.0556	158.97628/69.04472	1
Phosalone	11.06	367.99414	114.96150/138.01033	1	Indoxacarb	11.14	528.07799	203.01866/168.02107	1
Phosmet	9.98	318.00181	160.03930/133.02841	1	Methiocarb	10.14	226.08963	169.06816/121.06479	1
Phoxim	11.1	299.06138	129.04472/95.04945	1	Pentachlorophenol	11.74	262.83973	262.83973/262.83973	1
Pirimiphos-methyl	11.02	306.10358	108.05562/67.02907	1	Pirimicarb	8.38	239.15025	182.12879/72.04439	1
Prometryn	10.22	242.14339	200.09644/158.04949	1	Promecarb	10.29	208.13321	208.13321/109.06479	1
Propazine	10.1	230.1167	188.06975/146.02280	1	Propamocarb	4.25	189.15975	102.05496/74.02365	1
Pyrazophos	11.08	374.0934	194.05602/222.08732	1	Propiconazole	10.96	342.07706	158.97628/69.06988	1
Quinalphos	10.84	299.06138	147.05529/163.03245	1	Propoxur	8.89	210.11247	111.04406/95.04914	1
Simazine	9.01	202.0854	132.03230/124.08692	1	Robenidine	10.38	334.06208	138.01050/155.03705	1
Simetryne	9.13	214.11209	96.05562/124.08692	1	Thiobencarb	11.14	258.07139	258.07139/125.01525	1
Thiabendazole	7.26	202.04334	175.03245/131.06037	1	Thiophanate-ethyl	9.68	371.08422	151.03245/282.03654	1
Triazophos	10.42	314.07228	162.06619/114.96133	1	Thiophanate-methyl	8.85	343.05292	151.03245/311.02671	1
Tributyl-phosphorotrithioate	12.11	315.10344	57.06988/168.99052	1	Xylazine	7.26	221.1107	90.03720/164.05285	1
Trichlorfon	7.2	256.92985	127.01547/220.95318	1	2,3,5-Trimethacarb	9.56	194.11756	135.04406/107.04914	2
Doramectin	12.29	921.49708	777.41843/449.25097	2	3,4,5-Trimethylphenol	9.75	194.11756	135.04406/107.04914	2
Isoprocarb	9.56	194.11756	137.09609/95.04914	2	Phorate sulfone	9.55	293.00993	246.96807/114.96133	2
Methidathion	9.86	302.96913	145.00662/71.02399	2	Thiofanox sulphoxide	7.14	235.11109	57.06988/104.01646	2
XMC	9.39	180.10191	123.08044/113.99744	2	Avermectin B1a	12.23	895.48143	123.11683/153.05462	5
Aldicard	8.32	213.06682	95.04914/141.00593	5	Deltamethrin	12.04	521.00699	278.90762/89.05971	5
Dichlorvos	7.18	220.95318	127.01565/78.99452	5	Flucythrinate	11.73	469.19334	114.09134/199.0929	5
Propetamphos	10.34	282.09234	138.01370/156.02395	5	Flumethrin	12.33	532.0853	114.09134/73.02982	5
Thiofanox	9.56	241.09812	58.06513/184.07906	5	Ivermectin B1a	12.48	897.49708	753.41843/329.20872	5
Thiofanox sulphone	7.3	251.106	251.10600/57.06988	5	Methamidophos	2.57	142.00861	142.00861/112.01598	5
Bifenthrin	12.45	440.15987	181.10118/166.07770	10	Amitraz	11.97	294.19647	163.12298/122.09643	20
Phlorpyrifos-methyl	11.29	321.90226	142.99263/289.87605	10	Tau-fluvalinate	12.13	503.13438	181.06479/114.09134	20
Acephate	3.46	184.01918	113.00250/142.99263	20	Fenitrothion	10.18	278.02466	142.99263/149.02332	50
Aldicarb sulfoxide	4.78	207.07979	89.04195/69.05730	20	Methomyl	5.84	163.05357	88.02155/106.03211	50
Fenvalerate	12.05	437.16265	437.16265/114.09134	20	Phorate	11.11	261.0201	261.02010/75.02630	50
Validamycin	1.26	498.21812	142.08626/124.07569	50	Prothiofos	12.22	344.97009	258.92025/132.96046	100
Bromophos ethyl	12.21	392.8878	336.82520/161.96337	500	Cyfluthrin	12.01	451.0986	191.00250/114.09134	500
Parathion	10.86	292.04031	114.96133/138.00081	500					

## Supplementary Materials

The following is available online, Table S1: The detailed linear profile for 82 compounds.
