# Supplementary material for: Screening 89 Pesticides in Fishery Drugs by Ultrahigh Performance Liquid Chromatography Tandem Quadrupole-Orbitrap Mass Spectrometer
Source: Molecules. 2019 Sep 17;24(18):3375. doi: 10.3390/molecules24183375 (PMC6767809; doi:10.3390/molecules24183375)
Supplement: Supplementary file 1 [file molecules-24-03375-s001.pdf]

molecules

Article

Screening 89 Pesticides in Fishery Drugs by Ultrahigh Performance Liquid Chromatography Tandem Quadrupole-Orbitrap Mass Spectrometer

Shou-Ying Wang [1,2], Cong Kong [1,3*], Qing-Ping Chen [1,2] and Hui-Juan Yu [1,3*]

1 Laboratory of Quality & Safety Risk Assessment for Aquatic products (Shanghai), Ministry of Agriculture and Rural Affairs, East China Sea Fisheries Research Institute, Shanghai 200090, China; kongcong@gmail.com(C.K.); xdyh-7@163.com(H.J.Y.)

2 College of Food Science & Technology, Shanghai Ocean University, Shanghai 201306, China; magnolia7319@163.com (S.Y.W.); chenqp128@163.com (Q.P.C.)

3 Key Laboratory of East China Sea Fishery Resources Exploitation, Ministry of Agriculture and Rural Affairs, East China Sea Fisheries Research Institute, Chinese Academy of Fishery Sciences, Shanghai, China

* Correspondence: kongcong@gmail.com(C.K.); xdyh-7@163.com(H.J.Y.)

Table S1. The detailed linear profile for 82 compounds

Number	Compound	Linearity equation	R2	Linearity range(μg·kg^{-1})	SDL(mg/kg)
1	Chlorpyrifos	y=3598743.9x-66824597.7	0.9894	10-500	1
2	Phorate sulfoxide	y=5518870.1x+85085967.0	0.9979	1-500	1
3	Phoratoxon sulfoxide	y=7584503.7x+214355266.9	0.9918	1-500	1
4	Phosalone	y=4043085.9x+87273603.2	0.9917	1-500	1
5	Phoxim	y=20935952.6x+77732658.1	0.9968	1-500	1
6	Monocrotophos	y=3399617.5x+76912371.1	0.9953	1-500	1
7	Quinalphos	y=20935952.6x+77732658.1	0.9968	1-500	1
8	Dimethoate	y=3956848.5x+49609310.4	0.9990	1-500	1
9	Isocarbophos	y=322700.0x-3544966.5	0.9948	10-500	10
10	Omethoate	y=5644286.2x+109852302.6	0.9971	1-500	1
11	Coumaphos	y=12017548.2x-95154340.0	0.9954	1-500	1
12	Tributylphos-phorotrithioate	y=23130139.0x-227550662.9	0.9900	1-100	1
13	Mevinphos	y=1138089.3x+21492749.1	0.9935	10-500	1
14	Pyrazophos	y=14851456.7x+3576799.9	0.9969	1-500	1
15	Famphur	y=10429837.1x+239618543.1	0.9952	1-500	1
16	Pirimiphos-methyl	y=21984722.1x-76643690.4	0.9952	1-500	1
17	trichlorfon	y=2622329.4x+32875242.4	0.9987	1-500	1
18	Malathion	y=166327.7x-476935.3	0.9996	10-500	10
19	Triazophos	y=26439767.7x+444777130.2	0.9975	1-500	1
20	Phosmet	y=3115750.0x+41631055.0	0.9975	1-500	1

21	Prometryn	y=39008481.3x+1178898207.3	0.9989	1-200	1
22	Propazine	y=20602573.0x+740538113.2	0.9968	1-200	1
23	Simazine	y=13018037.4x+401259762.3	0.9910	1-500	1
24	Simetryne	y=31900235.6x+1041132510.4	0.9989	1-200	1
25	Thiabendazole	y=11969320.7x+697054283.8	0.9972	1-200	1
26	Fuberidazole	y=19923091.6x+1148557061.2	0.9943	1-200	1
27	Dodemorph	y=29301951.4x+917663702.2	0.9995	1-200	1
28	Imazalil	y=15562910.7x+500755563.5	0.9984	1-200	1
29	Carbendazim	y=9094262.0x+533734869.7	0.9945	1-200	1
30	Thiophanate-methyl	y=7646170.3x+489402526.7	0.9950	1-200	1
31	Thiophanate-ethyl	y=10200650.3x+512742750.7	0.9991	1-100	1
32	Propoxur	y=1037533.6x+32110226.9	0.9987	1-200	1
33	Carbaryl	y=13656425.2x+420652781.5	0.9907	1-500	1
34	Fenobucarb	y=5194113.2x+64068959.4	0.9972	1-500	1
35	Methiocarb	y=3245718.9x+83001666.2	0.9903	1-500	1
36	Promecarb	y=3350981.8x+91769603.5	0.9974	1-200	1
37	Pirimicarb	y=21646575.4x+723595624.1	0.9977	1-200	1
38	Aminocarb	y=8467394.6x+233011634.7	0.9905	1-500	1
39	Propamocarb	y=12944459.7x+292275449.2	0.9929	1-500	1
40	Carbofuran	y=9858716.2x+245568396.9	0.9925	1-500	1
41	Thiobencarb	y=2577085.7x+154150351.3	0.9994	1-100	1
42	Sodium pentachlorophenolate	y=500367.4x+7268421.1	0.9978	10-200	10
43	Chlordimeform	y=6952971.2x+121835191.3	0.9936	1-500	1
44	Indoxacarb	y=7568403.9x+137061737.0	0.9936	1-500	1
45	Propiconazole	y=49385699.4x+1800833099.2	0.9962	1-200	1
46	Robenidine	y=17016394.5x+376446884.1	0.9947	1-500	1
47	Xylazine	y=19945104.9x+457439572.1	0.9930	1-500	1
48	Ethoxyquin	y=15109557.7x+63329009.0	0.9986	1-500	1
49	Fipronil	y=3589275.4x+25270737.4	0.9975	1-500	1
50	Fipronil-sulfone	y=11699428.1x+184166622.7	0.9947	1-500	1
51	Fipronil-sulfide	y=7143758.4x+105783275.6	0.9945	1-500	1
52	Fipronil-desulfinyl	y=6861351.0x+104236245.4	0.9950	1-500	1
53	Aldicarb sulfone	y=2150114.6x+54113671.3	0.9902	1-500	1
54	Dioxacarb	y=883241.5x+36186523.6	0.9982	1-200	1
55	Bendiocarb	y=1753940.6x+57412241.9	0.9976	1-200	1
56	Phorate sulfone	y=2349959.5x+56472202.0	0.9925	1-500	1
57	Methidathion	y=1474288.5x+15350821.9	0.9983	10-500	1

58	Isoprocarb	y=8695896.2x+172635989.6	0.9927	10-500	10
59	XMC	y=1241370.0x+25003081.4	0.9938	10-500	10
60	Thiofanox -sulphoxide	y=257169.1x+8012535.0	0.9929	10-200	10
61	2,3,5-Trimethacarb	y=3869264.3x+43388801.3	0.9975	10-500	10
62	3,4,5-Trimethylphenol	y=4765480.4x+151927664.3	0.9965	10-200	10
63	Doramectin	y=1294667.1x-35413134.3	0.9954	1-500	10
64	Methamidophos	y=3639491.4x+49341388.7	0.9962	10-500	10
65	Propetamphos	y=324646.0x+3792202.1	0.9975	10-500	10
66	Aldicard	y=54857.0x+1256314.2	0.9941	50-500	10
67	Thiofanox	y=28955.5x+338209.0	0.9987	50-500	10
68	Thiofanox sulphone	y=341833.2x+11934640.7	0.9910	10-500	10
69	Dichlorvos	y=746370.1x+7477587.9	0.9993	10-500	10
70	Avermectin B1a	y=1403101.0x-44917519.5	0.9953	10-200	10
71	Ivermectin B1a	y=1433269.4x-35844660.8	0.9967	10-500	10
72	Flucythrinate	y=3732072.7x-137314047.8	0.9897	20-500	10
73	Deltamethrin	y=1419916.5x-78980510.6	0.9826	20-500	10
74	Flumethrin	y=1015405.8x-57818295.0	0.9849	50-500	10
75	Chlorpyrifos-methyl	y=558116.8x-6933601.7	0.9946	10-500	10
76	Aldicarb sulfoxide	y=281361.1x+8209821.0	0.9988	20-200	10
77	Fenvalerate	y=611595.8x-33130893.1	0.9903	50-500	10
78	Methomyl	y=76565.2x+2233236.4	0.9952	50-500	10
79	Tau-fluvalinate	y=653625.6x-39172597.6	0.9839	50-500	20
80	Amitraz	y=80394.3x-890600.1	0.9999	20-500	50
81	Fenitrothion	y=64528.4x-1618538.7	0.9984	50-500	50
82	Validamycin	y=130701.8x-285866.4	0.9992	50-500	50
